# Dengue virus transovarial transmission detection in *Aedes aegypti* from dengue hemorrhagic fever patients’ residences in Denpasar, Bali

**DOI:** 10.14202/vetworld.2022.1149-1153

**Published:** 2022-04-30

**Authors:** I. Made Sudarmaja, I. Kadek Swastika, Luh Putu Eka Diarthini, I. Putu Dema Prasetya, I. Md. Ady Wirawan

**Affiliations:** 1 Department of Parasitology, Faculty of Medicine, Udayana University, Bali, Indonesia; 2 Bachelor of Medicine Program, Faculty of Medicine, Udayana University, Bali, Indonesia; 3 Department of Public Health and Preventive Medicine, Faculty of Medicine, Udayana University, Bali, Indonesia

**Keywords:** *Aedes aegypti*, dengue virus, immunocytochemical, transovarial transmission

## Abstract

**Background and Aim:**

To effectively control dengue hemorrhagic fever (DHF), it is necessary to assess the risk of vertical virus transmission in *Aedes aegypti* mosquitoes. This study aimed to detect dengue virus (DENV) transovarial transmission in *A. aegypti* collected from DHF patients’ residences in Denpasar, Bali.

**Materials and Methods:**

*A. aegypti* samples were acquired by rearing *A. aegypti* eggs collected from ovitraps placed in the homes of DHF patients. Ovitraps were installed for 7 days and viewed using a loupe to determine whether there were *Aedes* spp. eggs present. An immunocytochemical method was utilized with 200 samples, and virus detection was performed using a reverse transcriptase-polymerase chain reaction (RT-PCR).

**Results:**

Of the 10 DHF patient houses fitted with ovitraps, four produced positive ovitraps from which larvae developed (house index=40%). Of the 50 ovitraps mounted in the 10 homes, 14 ovitraps were positive and contained *A. aegypti* eggs (ovitrap index=28%). Of these 14 positive ovitraps containing *A. aegypti* eggs, 10 ovitraps produced larvae. Immunocytochemical tests were conducted on *A. aegypti* eggs from the four houses under study. It was found that from the 200 samples collected, 197 samples could be observed, and 11 samples (5.6%) were positive for DENV antigen. RT-PCR examination conducted on mosquitoes reared from the four houses studied obtained a negative virus content result.

**Conclusion:**

This study found the presence of DENV antigen to be as high as 5.6%. This means that potential for transovarial transmission exists within DHF patients’ homes in Denpasar, Bali. *Aedes* control strategy in Denpasar should address this finding, in addition to the current approaches which have focused primarily on the elimination of larval breeding habitats and control of adults using insecticidal fogging during outbreaks.

## Introduction

Dengue hemorrhagic fever (DHF) remains a global health problem, especially for people living in tropical regions, including Indonesia [1], where dengue fever is the most common cause of hospitalization due to acute febrile illness in the country [2]. A recent analysis of National Disease Surveillance data spanning five decades indicated that the incidence rate (IR) of DHF appears to be cyclical, peaking approximately every 6-8 years, with the latest IR estimated at approximately 22.55 cases per 100,000 person-years [3]. As the most populous country in dengue-endemic Southeast Asia, Indonesia has consistently ranked among the top three countries with the most dengue cases [4]. Bali, a province of Indonesia, has historically high DHF IR and is almost always in the top five of all Indonesian provinces. DHF in Bali is endemic in nine districts with uneven distribution, with most cases occurring in Denpasar City, Badung Regency, and Gianyar Regency. In 2015, the number of DHF cases in Bali Province was 10,759, resulting in 29 deaths (IR=259.1/100,000 population), with especially high caseloads found in Denpasar City and Gianyar and Badung Regencies. The IR of DHF in Denpasar City reached 317.7/100,000 population in 2016 and continued throughout the year [2,5]. In 2019, the IR of DHF in Bali was 276.3/100,000 population and in Denpasar City was 155.88/100,000 population [6]. The emergence of new cases throughout the year is certainly strongly associated with the existence of vectors that remain active throughout the year and with dengue virus (DENV) within the vectors that also exist throughout the year.

The species of mosquito known to have the role as the main vector of DHF transmission is *Aedes* *aegypti* [7,8]. *Aedes* spp. mosquito fauna studies in Denpasar have also found *A. aegypti* to be the dominant species locally [9]. Specific drugs and effective vaccines for DHF are still under investigation [10]; therefore, the prevention of DHF has, until now, been mainly targeted at controlling its vectors, especially *A. aegypti* [11]. The government of Denpasar in Bali Province has made various efforts to control *A. aegypti* mosquito and reduce the prevalence of DHF. Still, it has not succeeded because its focus is solely on the horizontal transmission of DENV where *A. aegypti* mosquitoes are infected with DENV from feeding on DHF patients. Therefore, its efforts are oriented toward horizontal transmission-related actions, such as fogging when a new case is identified [5]. Some studies have shown that *A. aegypti* mosquitoes can contain viruses without ever feeding on a sick person but through the acquisition of the virus from their mother (transovarial). *A. aegypti* mosquitoes can become DHF transmitters from birth [12,13], so to carry out effective DHF control in Denpasar City, it is necessary to evaluate the possibility of virus transmission from *A. aegypti* mosquitoes vertically through transovarial transmission (TOT). It will then be possible to design a new *A. aegypti* eradication model, which is likely to be different from what is already in place. To develop a new response technique, research must determine whether the transmission of DENV transovarially from mosquito female to offspring is found in *A. aegypti* mosquitoes in Denpasar, Bali.

This study aimed to detect TOT of DENV in *A. aegypti* mosquitoes in Denpasar City and specifically assessed evidence of DENV infection and its prevalence in *A. aegypti* mosquitoes in Denpasar City. *Aedes* control strategy in Denpasar currently focuses primarily on the elimination of larval breeding habitats and the control of adults using insecticidal fogging during outbreaks. In Denpasar, there remains only limited evidence of TOT. New data will provide a greater understanding of the field interactions between DENV and its vectors and their links to human disease. These data can then aid in the establishment of reliable vector population target levels for commencing viral control and reduction strategies.

## Materials and Methods

### Ethical approval

Ethical committee exemption was obtained from the Head of the Department of Parasitology, Faculty of Medicine, Udayana University, because the study only involved invertebrates.

### Study period and location

The study was conducted from September to December 2019. The study was conducted in the territory of Denpasar City, Bali Province, Indonesia

### Collection of samples

*A. aegypti* samples were obtained by rearing *A. aegypti* eggs obtained from ovitraps installed in the homes of patients with DHF in Denpasar. Ovitraps were installed for 7 days and viewed using a loupe to determine whether there were *Aedes* spp. eggs present. The eggs were then soaked in water and the hatching larvae were allowed to develop until new pupae were transferred to a mosquito cage. The mosquitoes in the cage were fed 10% glucose until they reached 3 weeks old. They were then ready for examination for DENV within the mosquito bodies. The required number of samples for immunocytochemical examination was 200.

### Immunocytochemical examination [14,15]

The squash head preparation was done by squeezing the heads of *Aedes* spp. mosquitoes that had been turned and separated from their bodies and placed on an object-glass. Preparation was done individually. The preparations were immersed in a peroxidase blocking solution (PBS) (Sigma, USA) at 25-28°C for 10 min and then washed with PBS 3 times each for 2 min. Serum blocking solution (Sigma) was used for incubation at 25-28°C for 10 min, followed by incubation using 1C7 antibody (1:50) for 30-60 min using a damp incubator tray. Washing was performed with PBS 2 times each for 2 min. Incubation used streptavidin peroxidase conjugate for 5-10 min. After that, washing was again performed 3 times using PBS. Incubation continued using a mixture of chromogen substrate for 5-10 min and then was washed with Aquadest (Sigma). Incubation continued again with hematoxylin (Sigma) for 1-3 min and then a wash with tap water. The preparations were then washed with Aquadest, immersed in alcohol, and subsequently cleaned and immersed in xylol (Merck, Germany). Before examining under a microscope (Olympus, Japan), the preparations were placed on mounting media and covered with a coverslip and allowed to dry. Preparations indicating a brown color signified a positive result for the presence of DENV antigen, while either blue or pale indicated a negative result. In this study, both negative controls and positive controls were provided. Positive controls were infectious dengue mosquitoes that were reacted with primary antibodies. Negative controls were noninfectious preparations that were treated with primary antibodies.

## Results

Data on the presence of dengue fever patients who lived in Denpasar were retrieved from hospital information and the Denpasar Health Office. Of the 10 patients recorded, each of their houses was fitted with five ovitrap units so that a total of 50 ovitraps were installed. Of these 50 ovitraps, only 14 positive ovitraps containing *Aedes* spp. eggs were obtained. Ovitrap distribution is shown in Table-1.

**Table 1:** Distribution of positive ovitrap that contains *Aedes* spp. eggs.

Sample no.	Number of ovitrap	Ovitrap positive	Percentage	Develop into larvae
1	5	5	100	+
2	5	1	20	−
3	5	2	40	+
4	5	1	20	−
5	5	0	0	−
6	5	1	20	−
7	5	0	0	−
8	5	0	0	−
9	5	2	40	+
10	5	2	40	+
Total	50	14	28	4

The ovitrap index was found to be 28%, which means that the risk of transmission is still high in proximity to DHF patients. When viewed based on the eggs obtained from the 10 houses studied, seven houses had ovitraps which contained *Aedes* spp. eggs, but only four houses had eggs that developed into *A. aegypti* larvae (house index=40%). The high house index in this study does not imply that the potential for DHF transmission is necessarily so high, because the study intentionally provided ovitraps (containers) which were used by *A. aegypti* as breeding places, thus increasing the index artificially. Other containers used as breeding places (in addition to ovitraps) are, of course, not widely available because the Denpasar City government routinely commissioned a health worker (Jumantik) to come to people’s homes to control and eliminate breeding places in the homes [3,5].

### Detection of DENV antigen using immunocytochemical test

**Figure-1:**
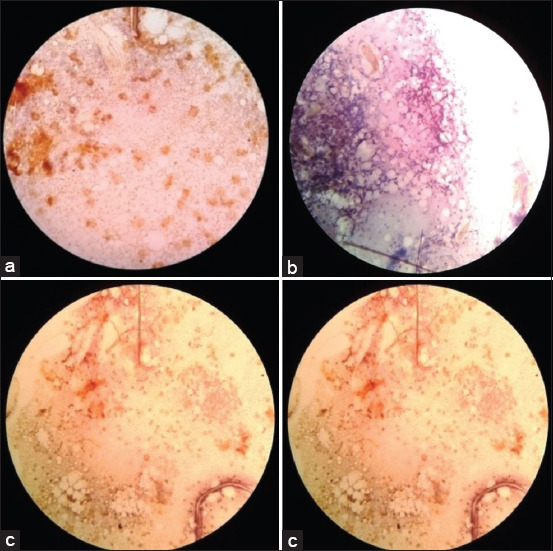
(a) Positive control, (b) negative control, and (c) positive samples.

Of the 200 squash head samples from randomly selected imago stage mosquitoes derived from four positive *A. aegypti* homes, only 197 samples were observed, because three samples were taken off the preparations. Of the 197 preparations observed, 11 positive preparations (5.6%) were found to show brownish in color. A 5.6% positivity rate shows that the potential for TOT occurs in *A. aegypti* mosquitoes in Denpasar City. The results of various positive preparations, positive controls, and negative controls are shown in Figure-1.

## Discussion

This study found that the presence of DENV antigen reached as high as 5.6%, which means there is a potential for TOT within DHF patients’ homes in Denpasar City. When the results of this study were compared with the results of the study in several other locations in Indonesia, this figure was found to be similar to studies conducted in several areas in four regencies in Central Java. In these areas, the detection of the DENV antigen using the immunocytochemical technique ranged from 0 to 8.77% [16]. Compared with research conducted in Manado, North Sulawesi, Indonesia, the results obtained from this study are lower because that research obtained a transovarial transmission index (TTI) of 12.2% using immunocytochemical examination [17]. Another study in the Malalayang District in Manado shows TTI from DENV in *A. aegypti* ranging from 6.1% to 17.1% [18]. Another study involving five cities involved in egg sampling showed that there were three cities with positive results of the equivalent of 60% of the proportion of transovarial events [16]. Another study found DENV infection through TOT in four serotypes. The most dominant stereotypes are DENV-4, DENV-3, DENV-1, and DENV-2 [17]. Another TOT study in Yogyakarta showed the opposite results, finding that only three stereotypes were detected (DENV-1, DENV-2, and DENV-3), while TOT was not found in DENV-4 [19].

The presence or absence of TOT can be determined through immunocytochemical techniques. The immunohistochemistry results in Figure-1 show that the dengue antigen was detected as a brown color in the cytoplasm of infected cells or as brownish granular deposits scattered in positive controls and positive samples. Meanwhile, negative results are shown as blue or purple in the negative control and negative samples. The results of this study were supported by the results of the examination of the DENV-3 antigen using the immunocytochemical method on the head squash preparation of *A. aegypti*, which showed positive results with varying degrees of transovarial infection. There were 33.33% (10 of 30) positive samples of DENV antigen in 4-day-old mosquito samples.

Meanwhile, 40% (12 of 30) samples were positive for DENV antigen in 16-day-old mosquito samples [20]. Another study showed the presence of DENV antigen in *A. aegypti* mosquitoes due to egg stage colonization taken from the residences of four DHF cases in Kombos Barat Village, Singkil District, Manado City, using immunohistochemical techniques. DENV detection results showed that *A. aegypti* mosquitoes which were positive for DENV constituted 24 of the 48 mosquitoes examined, with the average TTI of the four cases being 53.6% (39.1-70%) [21].

The mechanism of TOT in dengue can occur through three processes, namely, (i) uninfected female mosquitoes engage in sexual activity with transovarially infected male mosquitoes, resulting in sexual transmission of infection to female mosquitoes; (ii) uninfected female mosquitoes bite and suck a host’s viremic blood so that the virus is introduced and replicates in the mosquito’s body. Then, infected eggs are produced and develop into infected larvae; (iii) the ovarian tissue of female mosquitoes infected with the virus is passed down genetically to the next generation [22]. TOT was found more often inside houses than outside the house or in the yard (17 journals). These results contradict other studies showing that the ovitrap index (OI) in DHF endemic areas is greater outdoors than indoors. This finding is supported by a study in the Jakarta area which found that OI was greater in outdoor areas (36.4%) than indoor areas (33.5%). This is influenced by the behavior of *A. aegypti*, which prefers to lay eggs outdoors [18].

TOT is also more common in the summer than in the rainy season. TOT is said to increase gradually in the summer and peak in April-June. Meanwhile, dengue cases generally peak a month after entering the rainy season, namely, in September. Therefore, mosquito infections due to TOT generally occur in the 4 months before the incidence of DHF increases in humans [17]. Besides being influenced by the season and location of houses, the results of the previous studies showed that the presence of DENV was also influenced by road density, population density, and urban villages [23]. The finding of DENV TOT can cause an increase in dengue fever outbreaks and contribute to the persistence of dengue cases in endemic areas [22].

DENV TOT causes an area to remain endemic because cases will always exist and transmission will remain uninterrupted. A study showed that DENV TOT will experience a continuous increase in frequency until the seventh generation. Another study showed that there was a significant relationship between TOT and the incidence of DHF in Grogol District (p<0.001) [24]. The findings of TOT in various regions of Indonesia, and related descriptions of influencing factors, are important results for health program holders and will facilitate the development of syndromic surveillance, especially by epidemiologists. These surveillance data will better anticipate spikes in DENV so that outbreaks can be prevented [16].

The results of this study have several limitations. First, there is a time constraint because DENV may not have been given sufficient time to replicate, so false-negative results were still possible. Second, *Aedes* larvae were collected only from homes willing to participate, so the sample was not representative of the overall dengue infection prevalence in Denpasar. Therefore, more data are needed to obtain representative results regarding transovarial dengue transmission in local vectors in the Denpasar area.

## Conclusion

*A. aegypti* obtained through rearing from DHF homes in Denpasar with immunocytochemical examination was found to contain 5.6% DENV antigen which means that there is potential TOT in *A. aegypti*. The limited number of DHF cases during the study period resulted in a limited number of houses being included in the study and influenced the representativeness of the samples. It is expected that the findings of this study will aid in the control of the dengue epidemic through the reduction of the number of dengue cases in the future. In Denpasar, the present *Aedes* control strategy focuses primarily on eliminating larval breeding areas and controlling adults through insecticidal fogging during epidemics. The discovery of TOT indicates field interactions between DENV and vectors and their linkages to human disease. This can help define accurate vector population target levels for starting viral control and reduction initiatives.

## Authors’ Contributions

IMS: Devised the study’s concept and design. IMS, IKS, LPED, and IPDP: Assisted in sample collection, immunocytochemical examination, and data analysis. IMS and IMAW: Drafted, reviewed, and edited the manuscript. All authors have read and approved the final manuscript.
